# *RASSF10* is frequently epigenetically inactivated in kidney cancer and its knockout promotes neoplasia in cancer prone mice

**DOI:** 10.1038/s41388-020-1195-6

**Published:** 2020-02-11

**Authors:** Antje M. Richter, Michelle L. Woods, Miriam M. Küster, Sara K. Walesch, Thomas Braun, Thomas Boettger, Reinhard H. Dammann

**Affiliations:** 1 Institute for Genetics, University of Giessen, 35392 Giessen, Germany; 2 Max-Planck-Institute for Heart and Lung Research, Bad Nauheim, Germany; 3 German Center for Lung Research (DZL), Universities of Giessen and Marburg Lung Center, 35392 Giessen, Germany

**Keywords:** Cancer genetics, Renal cell carcinoma, DNA methylation

## Abstract

Kidney cancer incidences are rising globally, thereby fueling the demand for targeted therapies and precision medicine. In our previous work, we have identified and characterized the Ras-Association Domain Family encoding ten members that are often aberrantly expressed in human cancers. In this study, we created and analyzed the *Rassf10* knockout mice. Here we show that *Rassf10* haploinsufficiency promotes neoplasia formation in two established mouse cancer models (Rassf1A^−/−^ and p53^−/−^). Haploinsufficient *Rassf10* knockout mice were significantly prone to various diseases including lymphoma (Rassf1A^−/−^ background) and thymoma (p53^−/−^ background). Especially Rassf10^−/−^ and p53-deficient mice exhibited threefold increased rates of kidney cysts compared with p53^−/−^ controls. Moreover, we observed that in human kidney cancer, *RASSF10* is frequently epigenetically inactivated by its CpG island promoter hypermethylation. Primary tumors of renal clear cell and papillary cell carcinoma confirmed that *RASSF10* methylation is associated with decreased expression in comparison to normal kidney tissue. In independent data sets, we could validate that *RASSF10* inactivation clinically correlated with decreased survival and with progressed disease state of kidney cancer patients and polycystic kidney size. Functionally, we revealed that the loss of *Rassf10* was significantly associated with upregulation of KRAS signaling and *MYC* expression. In summary, we could show that *Rassf10* functions as a haploinsufficient tumor suppressor. In combination with other markers, *RASSF10* silencing can serve as diagnostic and prognostic cancer biomarker in kidney diseases.

## Introduction

Cancer still is a leading cause of morbidity and mortality worldwide. Fourteen million new cases per year, more than 8 million deaths, an economic impact above US$1 trillion and numbers are expected to rise further in the next years [1]. Expectedly, there has been a great scientific interest in the underlying genetic changes of cancer development, in the hope to decelerate these rising cancer numbers by targeted approaches. Biomarkers are of use at all disease stages and have an impact on most cancer patients’ diagnosis/treatment nowadays [2, 3]. Biomarkers like BRCA and HER-2 in breast cancer [4], PSA in prostate cancer [5] and EGFR in lung cancer [6] are already being valued in the clinic. Our group focuses on epigenetically inactivated tumor suppressors as candidate biomarkers [7–13], which are predicted to play a prominent role in the near future [14]. The tumor-suppressor RASSF10 is a member of the tumor-suppressor family Ras-Association Domain Family (RASSF) [15, 16]. The family has gained attention since its first description in the year 2000 [7]. The RASSFs differ substantially in their tumor-suppressor pathways [15–17]. RASSF10, contains its RA-domain N-terminally and lacks catalytically active domains [15, 16]. *RASSF10*, located at 11p15.3, contains a large CpG island promoter >2 kb (*NCBI, Entrez Gene*; Supplementary Fig. S1). Epigenetic inactivation of RASSF10 by its promoter hypermethylation is a frequent event in pathogenesis of human cancers [18–23]. In previous studies, we have shown that the *RASSF10* promoter is methylated in patient tumors samples of the adrenal gland [24], head and neck [20], sarcoma [20], pancreas carcinoma [20], and Merkel cell carcinoma [25]. We showed the epigenetic inactivation of *RASSF10* in breast cancer [26], lung cancer [20], skin cancer [27], and thyroid cancer [21] and showed that RASSF10 inhibited the growth of breast cancer [26], pancreas carcinoma, and sarcoma cell lines [20]. So far, RASSF10 has only been studied in vitro and inconsistent pathways of action were suggested like cAMP-PKA signaling [20], MMP2 [28], p53 [29], and JNK [30]. In vivo analyses of RASSF10 were missing and the function of RASSF10 has not been analyzed in kidney cancer.

In our study, we generated the first Rassf10 knockout mouse model/animal model and we present Rassf10 as a novel haploinsufficient tumor suppressor in vivo. The loss of Rassf10 is linked to upregulation of KRAS signaling and MYC induction. Moreover, we revealed that RASSF10 is frequently epigenetically inactivated in kidney cancer, and we show the clinical potential of RASSF10 as a biomarker in different kidney diseases.

## Results

### Generation and characterization of the Rassf10 knockout

**Fig. 1:**
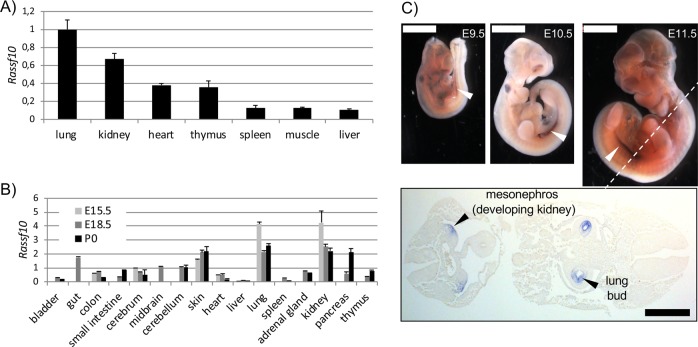
Rassf10 expression in mouse tissues. **a** RNA expression of *Rassf10* across mouse tissues from two adult wt C57BL/6 mice as normalized to *Gapdh* level (lung = 1). **b** *Rassf10* is expressed in the developing mouse shown for two embryonal stages (E15.5 and E18.5) and in newborn (P0). *Rassf10*-RNA levels are normalized to *Gapdh* expression. **c** *Rassf10* is expressed in whole mouse embryos by RNA in situ hybridization at E9.5, E10.5, and E11.5 (2 mm white bar) and according section is shown (1 mm black bar).

To analyze the tumor-suppressor function of RASSF10 in an animal model, we generated the Rassf10 knockout mouse. RASSF10 shares 86% amino acid identity between humans and mice regarding its sequence, domain structure (RA domain 87%) and the same genomic arrangement with a large CpG island covering its promoter (*NCBI*, *UCSC genome browser*, Supplementary Fig. S1). *Rassf10* is broadly expressed in adult wildtype mouse tissues (strain C57BL/6) and predominantly in lung, kidney, and thymus (Fig. 1a). During embryonal stages E15.5/E18.5 and newborn, we found that *Rassf10* is expressed in lung, kidney, and the developing skin (Fig. 1b). These expression data were verified by RNA in situ hybridization for *Rassf10* in embryos (E9.5/E10.5/E11.5; Supplementary Fig. 1C). According sections detected *Rassf10* in the developing kidney (mesonephros) and the lung bud/trachea. Due to Rassf10’s expression pattern, we generated a constitutive Rassf10 knockout (Supplementary Fig. S2). We interbred Rassf10^+/−^ animals (Fig. 1) and observed an alteration in the Mendelian ratio of the offspring (Table 1). The number of born Rassf10^−/−^ animals was significantly diminished (*p* < 0.04 *χ*^2^; Rassf10^−/−^ observed *n* = 67 vs. expected *n* = 87). We did not observe maldeveloped embryos (E14), implicating that Rassf10 loss impairs earlier embryogenesis. The surviving Rassf10^−/−^ animals developed normally (Supplementary Fig. S3) are fertile and showed no obvious phenotype.

**Table 1:** Mendelian ratio of Rassf10 knockout mice.

Rassf10 genotype^a^	Observed offspring			Expected offspring	Test *x*^2^
	Females	Males	Total		
+/+	52	46	98	87	*p* < 4.0E−02
+/−	82	101	183	174	
−/−	32	35	67	87	
Sum	166	182	348		

^a^From Rassf10^+/−^ P1 parental generation.

### The Rassf10 knockout promotes neoplasia in cancer prone mice

**Fig. 2:**
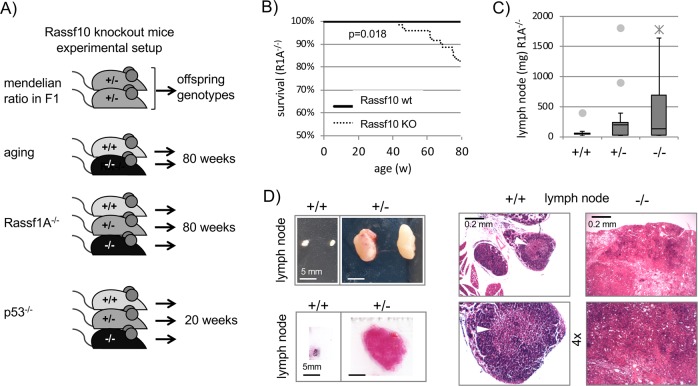
The Rassf10 knockout drives neoplasia in vivo. **a** Schematic representation of Rassf10 knockout mouse generation and experimental setup. Rassf10 knockout (KO) animals were subjected to aging, additional Rassf1A (R1A) knockout or p53 knockout. **b** Kaplan–Meier survival curves for Rassf10 KO mice reveal reduced survival vs. wt-Rassf10 animals in R1A^−/−^ background (*p* = 0.018, log rank test). Number of animals are 12 Rassf10^+/+^, 71 Rassf10^+/−^/Rassf10^−/−^. The *Y*-axis starting at 50%. **c** Rassf10 KO mice suffered from increased size of lymph nodes in comparison to wildtype Rassf10 mice in R1A^−/−^ background. Number of animals are 8 Rassf10^+/+^, 16 Rassf10^+/−^, and 24 Rassf10^−/−^. **d** The Rassf10 KO induces enlarged lymph nodes (lymphoma). Tissues were isolated during necropsy, fixed with formaldehyde, paraffin embedded, 10 µm sections were prepared and H&E stained before microscopic analysis.

We tested if Rassf10 loss induces spontaneous tumor formation in aging mice (Fig. 2a). At 80 weeks animals showed no differences between wt (*n* = 27) and Rassf10^−/−^ (*n* = 44), regarding the occurrence of various diseases/neoplasia (diseased wt 56% vs. Rassf10^−/−^ 57%). Therefore, we decided on a double knockout approach of tumor suppressors to accelerate disease/tumor onset, consistent with the cancer hypothesis of progressive accumulation of mutations [31]. We chose the p53^−/−^ (tumors <6 months [32, 33]) and separate Rassf1A^−/−^ (R1A; tumors at 18–20 months [34]) background to cross into our Rassf10 knockout (Fig. 2a). After 20 weeks for the p53^−/−^ double knockout and 80 weeks for the Rassf1A^−/−^ double knockout, all animals were sacrificed and full necropsy was performed (Table 2). In the Rassf1A^−/−^ tumor-suppressor background, the additional Rassf10 knockout reduced significantly the overall survival (*p* = 0.018; Fig. 2b). We observed significantly diseased animals Rassf10^+/−^ at 62% and Rassf10^−/−^ at 59% vs. wt at 24% (Table 2). In detail Rassf10 knockout animals mostly suffered from lymphoma, and the mean size of measurable lymph nodes increased from 86 mg for Rassf10 wt to 285 mg Rassf10^+/−^ to 401 mg for Rassf10^−/−^ (Fig. 2c, d). The median lymph node weight was 53, 201, and 136 mg, respectively. Lymph nodes were significantly enlarged in 53% Rassf10^+/−^ and 50% Rassf10^−/−^ vs. 10% Rassf10^+/+^ (>100 mg; Table 2). Rassf10 loss also enlarged the spleen and was present in 53% Rassf10^+/−^ and 40% Rassf10^−/−^ vs. 28% Rassf10^+/+^ (Table 2 and Supplementary Fig. S4; >95 mg). The mean spleen weight was 126 mg for Rassf10^+/+^ and increased by Rassf10 loss for Rassf10^+/−^ to 163 mg (Supplementary Fig. S4). The presence of lymphoma correlated with the enlargement of the spleen (splenomegaly; Supplementary Fig. S4B). In addition, Rassf1A knockout animals not only developed megaesophagus (reported earlier [35]), but also megaileum/megacolon at a total incidence 16%. This malformation of the digestive tract was Rassf10 independent and is a novel finding.

**Table 2:** Overview diseases Rassf10 knockout mice.

Systemic stress	Affected tissues	Rassf10 genotype	Diseased animals/total	Test *x*^2^
Rassf1A^−/−^ background	Diseased various	+/+	6/25 (24%)^a,b^	^a^*p* = 2.0E−03 ^b^*p* = 3.0E−03
		+/−	21/34 (62%)^a^	
		−/−	22/37 (59%)^b^	
	Lymphoma	+/+	2/21 (10%)^a,b^	^a^*p* = 1.7E−03 ^b^*p* = 1.9E−03
		+/−	9/17 (53%)^a^	
		−/−	11/22 (50%)^b^	
	Spleen	+/+	7/25 (28%)^a^	^a^*p* = 2.7E−02
		+/−	18/34 (53%)^a^	
		−/−	14/35 (40%)	
p53^−/−^ background	Diseased various	+/+	14/24 (58%)^a^	^a^*p* = 2.6E−02
		+/−	30/37 (81%)^a^	
		−/−	28/40 (70%)	
	Thymus	+/+	2/18 (11%)^a^	^a^*p* = 7.1E−03
		+/−	12/26 (46%)^a^	
		−/−	8/33 (24%)	
	Kidney	+/+	2/24 (8%)^a^	^a^*p* = 4.9E−02
		+/−	4/37 (11%)	
		−/−	10/40 (25%)^a^	

### The Rassf10 knockout promotes kidney neoplasia in p53-deficient mice

**Fig. 3:**
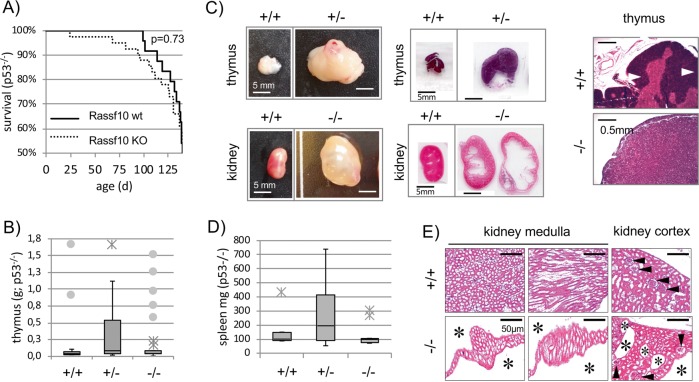
The Rassf10 knockout promotes neoplasia in p53-deficient mice. **a** Kaplan–Meier survival curves for Rassf10 knockout (KO) mice reveal reduced survival vs. wt- Rassf10 animals in p53^−/−^ background (not significant, log rank test). Number of animals are 24 Rassf10^+/+^, 77 Rassf10^+/−^/Rassf10^−/−^. **b** Heterozygous Rassf10 mice suffered from an increased thymus size/thymoma in comparison to wildtype Rassf10 mice in p53^−/−^ background. Number of animals are 18 Rassf10^+/+^, 26 Rassf10^+/−^, and 33 Rassf10^−/−^. **c** The Rassf10 KO induces enlarged thymus (thymoma) and induces cystic kidneys. Tissues were isolated during necropsy, fixed with formaldehyde, paraffin embedded, 10 µm sections were prepared and H&E stained before microscopic analysis. **d** Median spleen weight in p53^−/−^ background increases from Rassf10^+/+^ 100 mg (*n* = 5) to Rassf10^+/−^ 195 mg (*n* = 10; Rassf10^−/−^ 98.1 mg, *n* = 15). **e** Rassf10 knockout induces cystic kidneys in p53^−/−^ background and found in kidney medulla and cortex (10-μm sections, 20×). Cysts (asterisk) and glomeruli (►) are indicated. Fifty micrometers standard is shown.

In the p53^−/−^ tumor-suppressor background, survival analysis by Kaplan–Meier revealed that Rassf10 knockout animals (*n* = 77; Rassf10^+/−^/Rassf10^−/−^) had a reduced survival rate vs. Rassf10^+/+^ animals (*n* = 24, Fig. 3a, Table 2). However, this trend was not significant. We observed diseased animals at the following rates (p53^−/−^ background): wt 58% (14/24), Rassf10^+/−^ 81% (30/37), and Rassf10^−/−^ 70% (28/40). In detail, we found that the thymus and the kidney were mostly affected by Rassf10 knockout (Fig. 3 and Table 2). Interestingly, enlarged thymus and thymoma were found in 24% Rassf10^−/−^ (8/33), in 46% Rassf10^+/−^ (12/26) but only in 11% Rassf10-wt (2/18) animals, significant regarding Rassf10^+/−^ vs. wt (Table 2; Fig. 3b, c). Enlarged thymus were determined by *a* > 2.5*x* weight increase above average wt thymus (47.5 ± 2.4 mg Rassf10^+/+^; corrected by two thymoma; Supplementary Fig. S5). Rassf10 heterozygous knockout animals exhibited a dramatic increase in thymus weight, which was less pronounced in Rassf10 double knockout animals (Fig. 3b; Supplementary Fig. S5). The mean thymus weight (all weighed thymus) increased by 87% heterozygous Rassf10 knockout animals (344.8 mg) compared with wildtyp (184.6 mg). In addition, we observed that the median spleen weight increased in Rassf10^+/−^ by 95% vs. Rassf10^+/+^ (Fig. 3d). We also found a more than threefold increased occurrence of kidney cysts in Rassf10 knockout (p53^−/−^) animals 25% Rassf10^−/−^, 11% Rassf10^+/−^ vs. 8% wt (Table 2 and Fig. 3c, e). Kidney cysts were found in the medulla and cortex, which led to a compression and the structural loss of adjacent tissue, as well as size increase of the kidney when multiple cysts were present (Fig. 3e). Kidney cysts are similar to cancer with uncontrolled growth, the loss of tissue structure/function and loss of apoptotic control [36] and can be regarded a neoplasia [37].

### RASSF10 is epigenetically inactivated in human kidney cancer

**Fig. 4:**
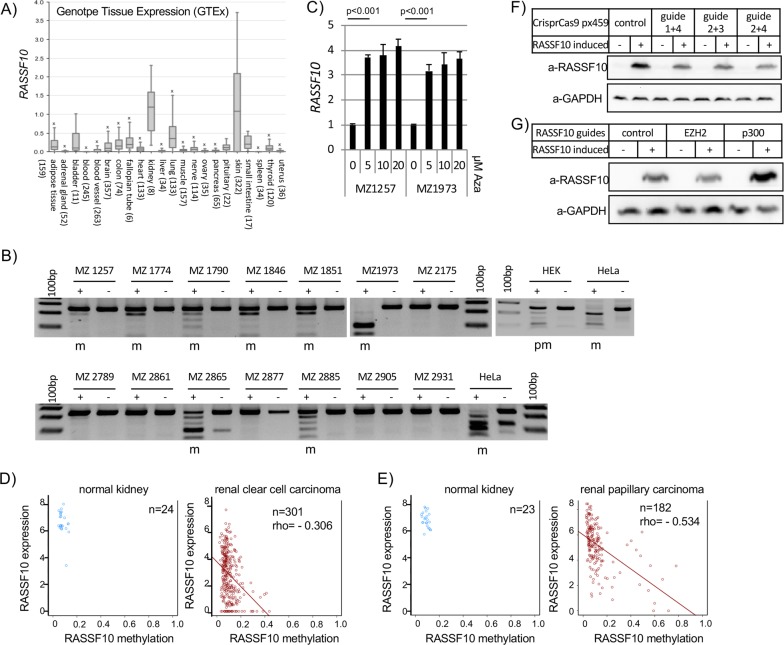
RASSF10 is epigenetically inactivated in human kidney cancer. **a** *RASSF10* expression across human normal tissues (log2, data GTEX—*n* = 2921—RPKM—ensgtexv4). **b** Kidney cancer cell lines were analyzed by combined bisulfite restriction analysis (+*Taq*I digested; −mock digest) for *RASSF10* promoter hypermethylation and 9 out of 15 (60%) were methylated (m). HeLa was used as positive control. HEK293 cell line is partially methylated (pm). **c** Pharmacological inhibition of DNA-methyl-transferases by 5-Aza-2′deoxycytidine (Aza) significantly reestablished *RASSF10* expression in kidney cancer cell lines MZ1257 and MZ1973 (*t*-test). *RASSF10* was normalized to *ß-ACTIN*. **d** In kidney cancer patient samples *RASSF10* methylation (cg05817758 in beta value) blocks *RASSF10* expression (log2; norm. rsem + 1) shown for renal clear cell and **e** renal papillary carcinoma. Analyzed using [79]. **f** RASSF10 inhibition by CRISPR-Cas9 genomic targeting of RASSF10. HEK293 (RASSF10-TetOn) cells were transfected with CRISPR-Cas9 guide RNAs in px549 vector targeting RASSF10 (three guide combinations) for 48 h and RASSF10 was induced by doxycycline for 24 h. **g** RASSF10 is epigenetically regulated by EZH2 and p300. HEK293 (RASSF10-TetOn) cells were transfected with RASSF10 guide RNAs in px549 (deadCas) targeting RASSF10 and recruiting EZH2(deadCas) or p300(deadCas) for 72 h and RASSF10 was induced by doxycycline for 24 h. Protein lysates were separated by SDS-Page and western blotted with indicated antibodies.

Tumor-suppressor inactivation in cancer can occur by the loss of function mutation or promoter methylation [38, 39]. *TP53* is heavily mutated in various cancers especially in lung (>80%), head and neck (>70%), colorectal (>50%), breast (>30%), kidney chromophobe (30%) and below 5% in kidney clear cell carcinoma [40]. For *RASSF10*, there are no reported mutations and *RASSF1* mutations are below 2% across primary cancers (Supplementary Table S1; TCGA; analyzed [41]). *RASSF10* contains a CpG island in its promoter region (Supplementary Fig. S1) and is expressed in normal human kidney, lung, skin, brain, and colon (Fig. 4a). Given the high *RASSF10* expression in the kidney and the affected kidneys upon Rassf10 knockout, we analyzed that the *RASSF10* promoter methylation in human kidney cancer. We found that *RASSF10* is epigenetically inactivated by promoter hypermethylation in 60% of kidney cancer cell lines (9/15, Fig. 4b). We could significantly reestablish the expression of *RASSF10* by DNMT inhibition treatment with 5-Aza-2′deoxycytidine in MZ1257 and MZ1973 (Fig. 4c). Primary tumors of renal clear cell and renal papillary cell carcinoma confirmed that *RASSF10* methylation correlated with decreased expression in comparison with normal kidney tissue (Fig. 4d, e). Using epigenetic editing by CRISPR-dCas9 system we were able to regulate RASSF10 expression (Fig. 4f, g). The targeted recruitment of epigenetic modulators/writers by the nuclease-deactivated-Cas9 to RASSF10 resulted in its expression modulation. Recruitment of histone acetyltransferase p300 activated RASSF10 and histone methyltransferase EZH2 reduced RASSF10 level in HEK293 cells (Fig. 4g). This result confirms the epigenetic regulation of RASSF10 in human kidney cells.

### RASSF10 inactivation correlates with clinical diagnosis and prognosis of human neoplasia of the kidney

**Fig. 5:**
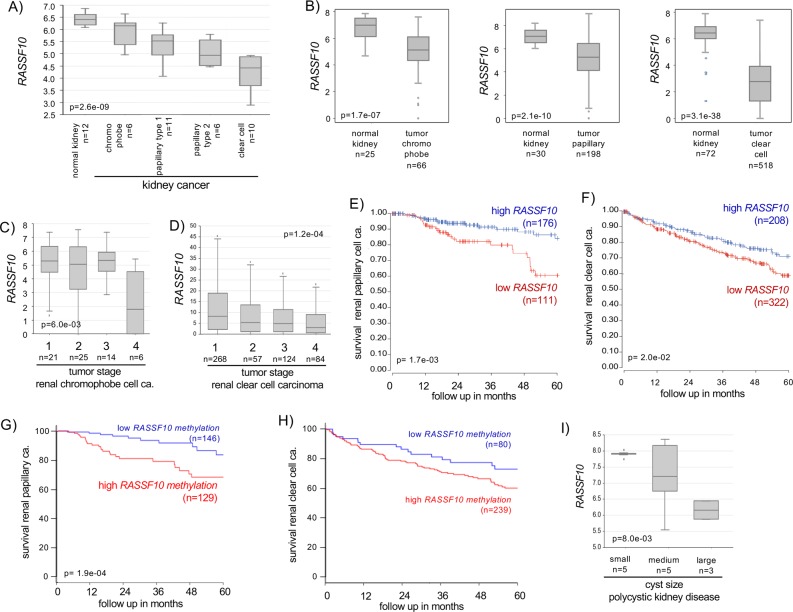
The loss of RASSF10 expression correlates with clinical diagnosis and prognosis of human neoplasia of the kidney. **a** RASSF10 expression is decreased in various kidney cancer types (log2, data Kort, Anova one way) and RASSF10 downregulation was verified in **b** chromophobe, papillary and clear cell renal carcinoma (log2, data TCGA, Wilcoxon). **c** The loss of RASSF10 expression correlated with kidney tumor stage in chromophobe cell carcinoma (log2, data TCGA, Anova one way) and **d** in clear cell carcinoma (data TCGA, Anova one way). **e** In renal papillary cell carcinoma and **f** renal clear cell carcinoma low RASSF10 levels correlated with reduced 5-year overall survival (data Pan-Cancer RNAseq). High RASSF10 methylation levels correlated with reduced survival in **g** renal papillary and **h** clear cell carcinoma (data TCGA). **i** RASSF10 levels are reduced with increasing cyst size in polycystic kidney disease (ADPKD, small cysts: <1 ml with *n* = 5 and each pool of 4; medium cysts: 10–25 ml with *n* = 5; large cysts > 50 ml with *n* = 3), (log2, data Pei, Anova one way).

Next, we tested the ability of RASSF10 to serve as a prognostic and diagnostic biomarker in independent data sets for human neoplasia across various primary samples. *RASSF10* expression is decreased in various types of kidney cancer (chromophobe, papillary, and clear cell, Fig. 5a), which was verified in a further data set and each was significant compared with normal kidney tissues (Fig. 5b). Reduced *RASSF10* expression correlated with progressed tumor stage in renal chromophobe and renal clear cell carcinoma (Fig. 5c, d). In renal carcinoma (papillary and clear cells) overall survival correlated with high *RASSF10* expression (Fig. 5e, f). We found that the low *RASSF10* methylation levels are favorable for kidney cancer survival (renal papillary and clear cell carcinoma, Fig. 5g, h). Since we observed in the Rassf1A^−/−^ and TP53^−/−^ mice background increased neoplasia for the Rassf10 knockout (Table 2), we analyzed overall survival rate in 288 renal papillary carcinomas with the low RASSF10 and RASSF1 or TP53 expression in renal papillary cell carcinoma [42] (Supplementary Fig. S6). We observed that the low RASSF10 and low RASSF1 expression was significantly associated with impaired survival (*p* = 0.018), however, this was not significant for the low TP53 expression (Fig. S6).

In polycystic kidney disease, there was a correlation between reduced *RASSF10* expression and an increasing cyst size (Fig. 5i), consistent with Rassf10 loss driven renal cysts in mice and the longstanding idea of polycystic kidney disease as a neoplasia in disguise [37]. In summary, we observed that the levels of *RASSF10* expression/methylation in combination with RASSF1A expression are suitable for prognosis and diagnosis of various kidney cancer types in humans.

### Upregulation of KRAS signaling and MYC are associated with the loss of RASSF10 and impaired survival

**Fig. 6:**
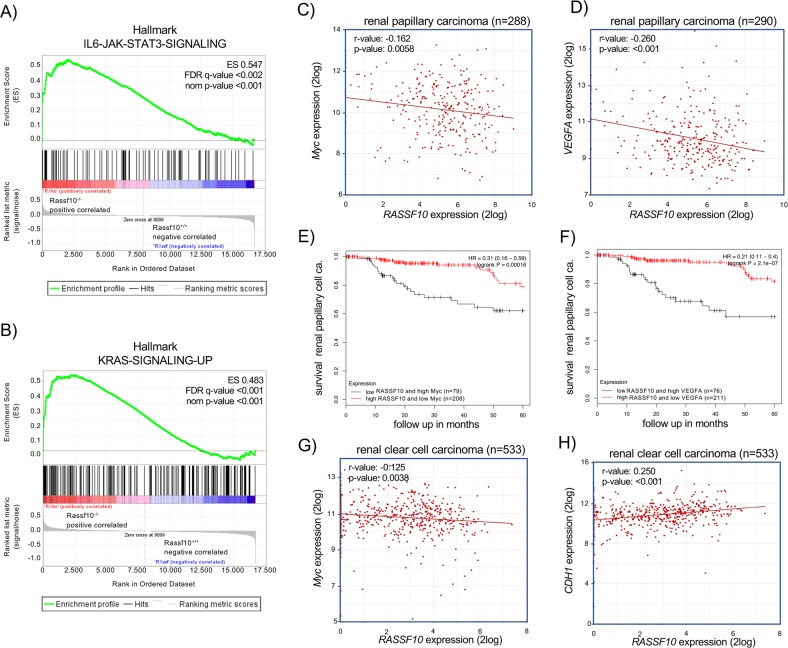
Upregulation of oncogenic signaling pathways are associated with inactivation of RASSF10 and impaired survival of renal carcinoma patients. RNA isolated from Rassf10^−/−^ and Rassf10 wildtype mouse embryonic fibroblasts were analyzed on MoGene-2_0-stRNA microarrays. Upregulated hallmarks were identified by gene set enrichment analysis. Enrichment plot for **a** IL6-JAK-STAT3 and **b** KRAS signaling are depicted. **c** *MYC* and **d** *VEGFA* are significantly negatively correlated with *RASSF10* expression in papillary cell carcinoma (TCGA data set). Mean expression of *RASSF10* and **e** *MYC* or **f** *VEGFA* in renal papillary cell carcinoma was analyzed in the pan-cancer RNA-seq panel with Kaplan–Meier plotter [42] and survival probability was plotted for low *RASSF10* and high *MYC/VEGF* (black) expressing and high *RASSF1*0 and low *MYC/VEGF* (red) expressing samples. **g** In clear cell carcinoma *MYC* is significantly negatively associated with *RASSF10* expression and **h** expression of *CDH1* is positively correlated with RASSF10. Correlation analysis of expression data using *R2 Genomics Analysis and Visualization Platform* [71] from human primary renal carcinomas (log2 data, TCGA data set).

To gain insight in the molecular processes that are deregulated upon Rassf10 inactivation we utilized mouse embryonic fibroblasts (MEFs) isolated from Rassf10 knockout and wildtype mice. Subsequently, we performed RNA microarray analysis to analyze the altered gene expression in knockout compared with wildtype MEFs. Gene ontology analysis of deregulated RNA levels was performed and this analysis shows a significant association with the molecular function of signaling receptor, molecular transducer and transmembrane signaling receptor activity (Table 3). Gene sets enrichment analysis showed a significant upregulation of IL6-JAK-STAT signaling and KRAS signaling in Rassf10 deficient MEFs compared with wildtype (Fig. 6a, b, respectively). Other hallmarks that were significantly associated the Rassf1a knockout belong to allograft rejection, inflammatory response, complement, and interferon gamma response gene sets. These results suggest that upon Rassf10 depletion several oncogenic pathways including RAS signaling are activated.

**Table 3:** Gene ontology analysis of Rassf10^−/−^ microarray.

Description	GO pathway annotation (molecular function)	*p* value	FDR *q* value
>1.4 × upregulated in Rassf10 knockout versus wildtyp	Signaling receptor activity GO:0038023	2.65E−14	9.08E−11
	Molecular transducer activity GO:0060089	2.48E−13	4.26E−10
	Transmembrane signaling receptor activity GO:0004888	2.64E−12	3.02E−09
<0.6 × downregulated in Rassf10 knockout versus wildtyp	Transmembrane signaling receptor activity GO:0004888	1.36E−30	4.67E−27
	Signaling receptor activity GO:0038023	1.86E−29	3.2E−26
	Molecular transducer activity GO:0060089	4.81E−28	5.51E−25

MYC is an oncogenic downstream target of KRAS and IL6-JAK-STAT signaling [43, 44] and therefore we correlated the expression of *MYC* and *RASSF10* in primary renal papillary cell carcinoma and clear cell carcinoma (Fig. 6c, g). In papillary cell carcinoma, we found a significant inverse correlation of *RASSF10* and *MYC* or *VEGF* expression (Fig. 6c, *p* < 0.006 and Fig. 6d, *p* < 0.001, respectively). VEGF is an *bona fide* target of IL6-JAK-STAT pathway involved in angiogenesis [45]. Furthermore, we correlated overall survival of renal papillary cell carcinoma patients with *RASSF10* and *MYC* or *VEGF* expression by Kaplan–Meier analysis (Fig. 6e, f). For both analysis a significant impaired survival was observed for low *RASSF10* and high *MYC* or *VEGF* expression compared with the high *RASSF10* and low *MYC* or *VEGF* (*p* = 1.6 × 10^−4^ and *p* = 2.1 × 10^−7^; Fig. 6e, f, respectively). In renal clear cell carcinoma, we also detected an inverse correlation between *RASSF10* and *MYC* expression indicating that the loss of *RASSF10* is associated with *MYC* induction (Fig. 6g, *p* < 0.004). Recently, it has been reported that RASSF10 regulates epithelial *cadherin* (CDH1) expression, that is a tumor suppressive TGFβ-target and inhibits epithelial-to-mesenchymal transition (EMT) [46]. Interestingly, we found that *RASSF10* expression is positively associated with increased *CDH1* levels in renal clear cell carcinoma (Fig. 6h; *p* = 4.6 × 10^−9^). Our data suggest that *MYC* and *VEGF* are negatively regulated by RASSF10 and that combination of high *RASSF10* and low *MYC* or *VEGF* expression is associated with a favorable prognosis for renal cancer patients.

## Discussion

We report for the first time that Rassf10 promotes tumor formation in vivo in a double knockout background with the p53 or Rassf1A tumor suppressors and this resulted in reduced survival and increased disease burden (Figs. 2 and 3). Rassf10 knockout promotes increased numbers of thymoma (p53^−/−^ [47]), cystic kidneys (p53^−/−^ [48, 49]), lymphoma (Rassf1A^−/−^ [34], p53^−/−^ [32, 50]), and splenomegaly (Rassf1A^−/−^, p53^−/−^ [51]). Rassf10 heterozygous animals already suffer from an overall increased disease incidence (Table 2), suggesting Rassf10 is a haploinsufficient tumor suppressor, and the loss of one allele is sufficient for its loss of function and contribution to carcinogenesis. The effects could be directly attributable to the reduction in gene dosage or may act in concert with other oncogenic or haploinsufficient events (like PTEN, p53 or p27) [52, 53]. The mouse genetic background has a strong impact on tumor susceptibility and is mouse strain dependent [54, 55]. We have chosen the well characterized and established in house strain C57BL/6 for which Rassf1A and p53 knockouts existed and confirmed expression of Rassf10 in different tissues, including kidney (Fig. 1). Given the relative tumor suppressive influence of the C57BL/6 background [55–57], one could anticipate to observe further tumor types upon Rassf10 loss, like we report here for human patients, in other mouse strains or in combination with other tumor suppressors or even in an oncogenic background (e.g., KRAS), which might be addressed by others in the future. As a tumor suppressor in vivo, we wanted to understand the RASSF10 mechanism of regulation and contribution to carcinogenesis. We reported that knockdown of RASSF10 increased mitosis and increased cell proliferation and invasion, as well as RASSF10 reexpression halted proliferation [20, 26, 46]. In addition, RASSF10 was found at centrosomes/microtubules during mitosis [19]. Interestingly, we found that the loss of Rassf10 in MEFs is associated with upregulation of KRAS and IL6/JAK/STAT3 signaling (Fig. 6). The alteration of these oncogenic pathways is hallmarks in human carcinogenesis and leads to activation of signaling receptor and molecular transducer pathways, which was detected in the Rassf10 knockout MEFs (Table 3). RASSF10 contains a N-terminal Ras-association domain, but it is direct interaction which Ras has been not reported. A RASSF member, which contains a C-terminal RA domain and is bound to Ras, is the novel Ras effector NORE1 (RASSF5) [58]. It is interesting to note that mutations of Ras oncogenes do not play a major role in the initiation and progression of renal carcinomas [59]. This suggests that the activation of KRAS signaling may be accomplished by the inactivation of RASSF10.

Across different renal cancer entities, we found a significant epigenetic inactivation of *RASSF10* (Figs. 4 and 5). In addition, we can exclude mutation events in the inactivation of *RASSF10* in cancer (Table S1). DNA hypermethylation of gene promoters in cancer is a well-established mechanism of tumor-suppressor inactivation [60]. We could clearly show that the loss of *RASSF10* expression in kidney cancer correlated with its increasing promoter methylation (Fig. 4). The successful pharmacological inhibition of DNA methylation restored *RASSF10* expression, which we reported earlier in other cancer entities [20, 21, 26, 27]. We suggest that for the inactivation of the haploinsufficient tumor-suppressor RASSF10 the methylation of one allele is sufficient and found that low methylation levels of *RASSF10* already decreased its expression dramatically. This is consistent with the observation that we neither found *RASSF10* mutations in cancer nor LOH for the *RASSF10* locus. Our clinical patient data revealed that the loss of *RASSF10* is a common and general event in kidney carcinogenesis (Fig. 5). We could also show that in independent data sets *RASSF10* loss correlated not only with reduced patient survival rates, but also with tumor stage/grade and was found in different kidney entities (clear cell carcinoma, papillary cell carcinoma, and chromophobe cell cancer). In addition, we observed that *RASSF10* downregulation significantly correlates with upregulation of *MYC* or *VEGF* levels (Fig. 6). MYC and VEGF are *bona fide* oncogenic downstream targets of KRAS and IL6/JAK/STAT3 signaling pathways [43–45]. Low *RASSF10* and high *MYC* or high *VEGF* was significantly associated with poor prognosis of renal papillary cell cancer patients and the combination of two markers had a higher impact on impaired probability of survival compared with the low *RASSF10* alone (Figs. 5e and 6). Moreover, we found that *RASSF10* expression is positively correlated with *E-cadherin* (CDH1) expression (Fig. 6). CDH1 is a tumor suppressive regulator of cell adhesion in epithelial cells and prevents EMT that is an important step in tumor invasion [61]. It has been reported that RASSF10 regulates *CDH1* expression through the *apoptosis stimulating protein of p53* (ASPP2) and inhibits TGFβ induced invasion of lung cancer cells [46].

Here we achieved the discovery and validation of the *RASSF10* biomarker across different kidney tumor types, which in the next steps of biomarker development will have to be followed by assay development and analytical validation, clinical utility validation, and ultimately clinical implementation [2]. There are several FDA-approved cancer biomarkers currently used in clinical practice for e.g. liver, prostate, ovarian, breast, pancreatic, lung, and thyroid cancer [62, 63] and a blood-based colorectal DNA methylation marker screening test [64]. An epigenetic assay for *RASSF1A* methylation in prostate cancer, however, still biopsy based, is available [65, 66]. We could show that epigenetic editing of RASSF10 is possible, and one could think of targeted therapies in the future using virally applied CRISPR-Cas9 Epigenetic Editors to patients for reactivation of e.g. hypermethylated RASSF10 specifically in cancer (Fig. 4). *RASSF10* is inactivated in various cancer types by DNA hypermethylation of its promoter. We are presenting compelling evidence that the haploinsufficient tumor-suppressor RASSF10 can be used as a prognostic and diagnostic cancer biomarker in combination with other tumor related genes (e.g., MYC) in kidney diseases.

## Materials and methods

### Rassf10 mouse

The bacterial artificial chromosome (BAC) backbone pBACe3.6 containing the single exon of *Rassf10* (Source BioScience) was equipped with homologous regions 5′ and 3′ to Rassf10 genomic sequence for homologous recombination. Rassf10 cassette was then inserted into pKO24 vector and altered to contain LoxP sites flanking the coding region of *Rassf10*, as well as a neomycin cassette 3′ of lower LoxP site for positive selection. Vector contained diphtheria-toxin A cassette for negative selection. Completed *Rassf10* knockout vector was used for homologous recombination in mouse embryonal stem cells of C57BL/6 mouse strain. Floxed *Rassf10* containing animals (verified by southern blotting) were interbred with Cre recombinase expressing animals and the deletion of *Rassf10* was verified by southern blotting, genomic PCR and on RNA expression levels.

### Rassf10 mouse experimental setup

Rassf10 heterozygous parental animals were interbred to determine altered mendelian ratio of offspring (*n* = 348). Rassf10 knockout and wildtype mice were analyzed for spontaneous tumor formation by aging (Rassf10^+/+^ *n* = 27, Rassf10^−/−^ *n* = 44). When reaching 80 weeks of age, mice were sacrificed and complete necropsy was performed. Rassf10 knockout animals were interbred with p53 or Rassf1A deficient animals and animals were obtained from Johnny Kim (Max-Planck Institute, Bad Nauheim, Germany, p53 knockout animals) and from Gerd Pfeifer [34] (City of Hope, CA, USA; Rassf1A knockout animals). Experimental setup in p53^−/−^ was 20 weeks and in Rassf1A^−/−^ was 80 weeks as published earlier and full necropsy was performed. Tissues were formaldehyde fixed, ethanol dehydrated, paraffin embedded, sectioned for analysis by H&E staining and microscopy. All experimental mouse work was approved by local authorities, animal care was in accordance with institutional guidelines and local authorities guidelines (Regierungspräsidium Darmstadt) and was performed at Max-Planck Institute, Bad Nauheim, Germany at Thomas Boettger and Thomas Braun laboratory.

### Southern blotting

Genomic DNA for genotyping was isolated and digested with *EcoR*I (5′ probe detection) and *BamH*I (3′ probe detection). Digestion products were separated in 0.8% TAE gel and blotted onto nylon membrane, which was hybridized with 5′/3′ probes at 65 °C and finally analyzed by phosphor imager. For Rassf10 probe generation: genomic Rassf10 flanking regions were amplified by PCR, cloned into pJet1.2/blunt (Thermo Fisher Scientific), *Bgl*II digested and extracted from agarose gel. Probes were then radioactively labeled by a-P [10]-dCTP incorporation (Rediprime DNA Labeling System, Amersham) and purified by MicroSpin G-50 columns (GE Healthcare).

### RNA in situ hybridization

At first mouse *Rassf10* was cloned into pCMVTag1, linearized by digestion with *ApaL*1, *Rassf10* was transcribed by T7 polymerase, and thereby *Rassf10*-RNA labeled with the RNA Labeling DIG Kit (Roche), and followed by Rassf10-RNA probe purification. C57BL/6 embryos were isolated, paraformaldehyde fixed, hybridized with Rassf10-RNA probe, and developed by BCIP/NBT (Sigma) [67, 68]. The stained embryos were then paraffin embedded and sectioned (10 µM) for analysis.

### DNA methylation analysis

Promoter region of *RASSF10* and Rassf10 was analyzed by CpG plot http://www.ebi.ac.uk/Tools/seqstats/emboss_cpgplot/ and both show CpG islands (Fig. S1). Primers for bisulfite treated DNA were designed to bind only fully converted DNA and amplify promoter region (Supplementary Table S2). DNA methylation of the RASSF10 promoter was analyzed by combined bisulfite restriction analysis (CoBRA) [21, 27]. The CoBRA PCR product for *RASSF10* is 241 bp (*Taq*I sites at 49 + 140) or for nested 167 bp (*Taq*I site at 66 bp). DNA was isolated after proteinase K (Thermo Fisher Scientific) digest and extracted either with phenol/chloroform or by QIAamp DNA extraction kit (Qiagen), and concentrations were determined by UV-photospectrometery. For COBRA methylation analysis, 2 µg genomic DNA was bisulfite treated (12 µl 0.1 M hydroquinone, 208 µl 1.9 M sodium metabisulfite, and pH 5.5 with NaOH) and incubated over night at 50 °C. DNA was purified using MSB Spin PCRapace (STRATEC Molecular), eluted in 50 µl H_2_O and followed by 10 min incubation with 5 µl 3 M NaOH at 37 °C. DNA was then precipitated with 100% ethanol and 7.5 M ammonium acetate and resolved in 1 × TE buffer. The subsequent PCR product was digested with 5 U of *Taq*I (Thermo Fisher Scientific) 1 h at 65 °C and resolved on 2% TBE gel together with mock control and DNA ladder. In vitro methylation of genomic DNA was performed using CpG Methyltransferase *M.Sss*I (NEB) according to manufacturer’s protocol.

### RNA expression analysis

RNA was isolated from human cell culture or mouse primary tissues (homogenized using Bioruptor, Diagenode) using Isol-RNA lysis procedure (Trizol, Thermo Fisher Scientific). RNA was *DNase* (Thermo Fisher Scientific) treated and then reversely transcribed by MMLV (Promega). Quantitative RT–PCR was performed in triplicate with SYBR select (Thermo Fisher Scientific) using Rotor-Gene 3000 (Qiagen). For primers see Supplementary Table S2. RNA microarrays (MoGene-2_0-st, Thermo Fisher Scientific) were performed according to manufacturer’s protocol (P/N 703174 Rev. 2) with 200 ng of total RNA. Reagents/equipments were GeneChip® WT PLUS Reagent Kit, P/N: 902280; GeneChip® Hybridization, Wash, and Stain Kit P/N 900720, GeneChip Scanner, GeneChip Fluidics Station 450, GeneChip Hybridization Oven 640, Bioanalyzer 2100 (Agilent) and RNA600 NanoKit (Agilent). Microarray data are available in the ArrayExpress database (www.ebi.ac.uk) under accession number E-MTAB-8296.

### Cell culture and treatment of cell lines

Cell lines were grown in appropriate medium (DMEM, RPMI) supplemented with 10% FCS, 1% penicillin/streptomycin under cell culture conditions (37 °C, 5% CO2). For 5-Aza-2′-deoxycytidine (Aza) treatment cells were split to 10% density and Aza was added with fresh medium on 4 consecutive days at working concentrations of 5, 10, and 20 µM before cell isolation. Cell lines were transfected for indicated time points using Polyethylenimmin (PEI, 4,9 mM, Sigma) for HEK293 cells. Doxycycline (Dox, Thermo Fisher Scientific) was dissolved in water and used for RASSF10 induction in HEK293 cells within the TetOn-TREx-system at working concentration of 2 µg/ml for 48 h [69].

### Generation of stable RASSF10 inducible HEK293 cells

HEK293 stably expressing the Tet repressor under Blasticidine (5 µg/ml, Roth) were obtained from Thermo Fisher Scientific as a part of the TetOn-TREx-System, and were used as control cell line. These cells were transfected with the cloned RASSF10-pcDNA4ToMyc for stable insertion of Dox-inducible-RASSF10. Cells were selected for RASSF10 using Zeocin (500 µg/ml, Thermo Fisher Scientific).

### Western blotting

Proteins were separated via SDS-PAGE and western blotted onto PVDF membrane (Immobilon) for antibody-based detection. Luminata Crescendo Western HRP substrate (Millipore) was used for detection at VersaDoc Imaging System (BioRad). The following antibodies were used: a-GAPDH (FL335, sc-25778 from Santa Cruz), a-RASSF10 (AP12444c-ev2020, Abgent), and HRP-coupled secondary antibodies anti-rabbit (sc2004, sc2357, Santa Cruz).

### Genomic and epigenetic editing CRISPR-Cas9

To genomically delete *RASSF10*, we performed CRISPR-Cas9 targeted knockout. CRISPR-Cas9 vectors were obtained from Lienhard Schmitz (Giessen, Germany) and *RASSF10* targeting oligos/guide RNAs were generated according to protocol [70]. Four *RASSF10* knockout oligos were created to generate a frameshift and RASSF10 loss (three combinations) in px549 with wt-Cas9 (Supplementary Table S3). We transfected A549-cells with the CRISPR-Cas9 RASSF10 oligos and selected for positive clones by puromycin (1 µg/ml) for 3 days. Clones were expanded and the knockout was verified by PCR based amplification of the *RASSF10* genomic region and RNA showing the deletion and was further verified by western blot. For the epigenetic editing of RASSF10, we created a deactivated Cas9 (px549dCas, by deletion) and cloned the RASSF10 targeting guide RNAs. EZH2 (Ezh2[SET]-dCas9 Addgene) and p300 (pcDNA-dCas9-p300 Core Addgene) were used as epigenetic modifiers upon cotransfection in HEK293 with guide RNAs(dCas).

### Statistical analysis

Gene expression, promoter methylation correlation, and Kaplan–Meier calculations were performed using *R2 Genomics Analysis and Visualization Platform* [71], *Wanderer* [72]*, KM Plotter* [42, 73–75], and *MethSurv* [76]. Gene ontology analysis of microarray data of differentially expressed genes (DEGs; TGFß DEG according to [77, 78]) was performed using *Gene Ontology enRIchment anaLysis and visuaLizAtion tool Gorilla* [79]. For further calculations, we used *GraphPad* (https://www.graphpad.com/quickcalcs/). Mendelian ratio alteration was determined by Chi square test. Knockout mice disease proneness was calculated by Fisher Exact Test. Student’s *t* test (unpaired; two tailed) was used for reexpression of RASSF10 under Aza treatment.

## Supplementary information

Supplemental Figures and Tables

Supplementary datasets
