# Supplementary material for: RASSF10 is frequently epigenetically inactivated in kidney cancer and its knockout promotes neoplasia in cancer prone mice
Source: Oncogene. 2020 Feb 11;39(15):3114–27. doi: 10.1038/s41388-020-1195-6 (PMC7142015; doi:10.1038/s41388-020-1195-6)
Supplement: Supplementary file 1 — Supplemental Figures and Tables [file 41388_2020_1195_MOESM1_ESM.pdf]

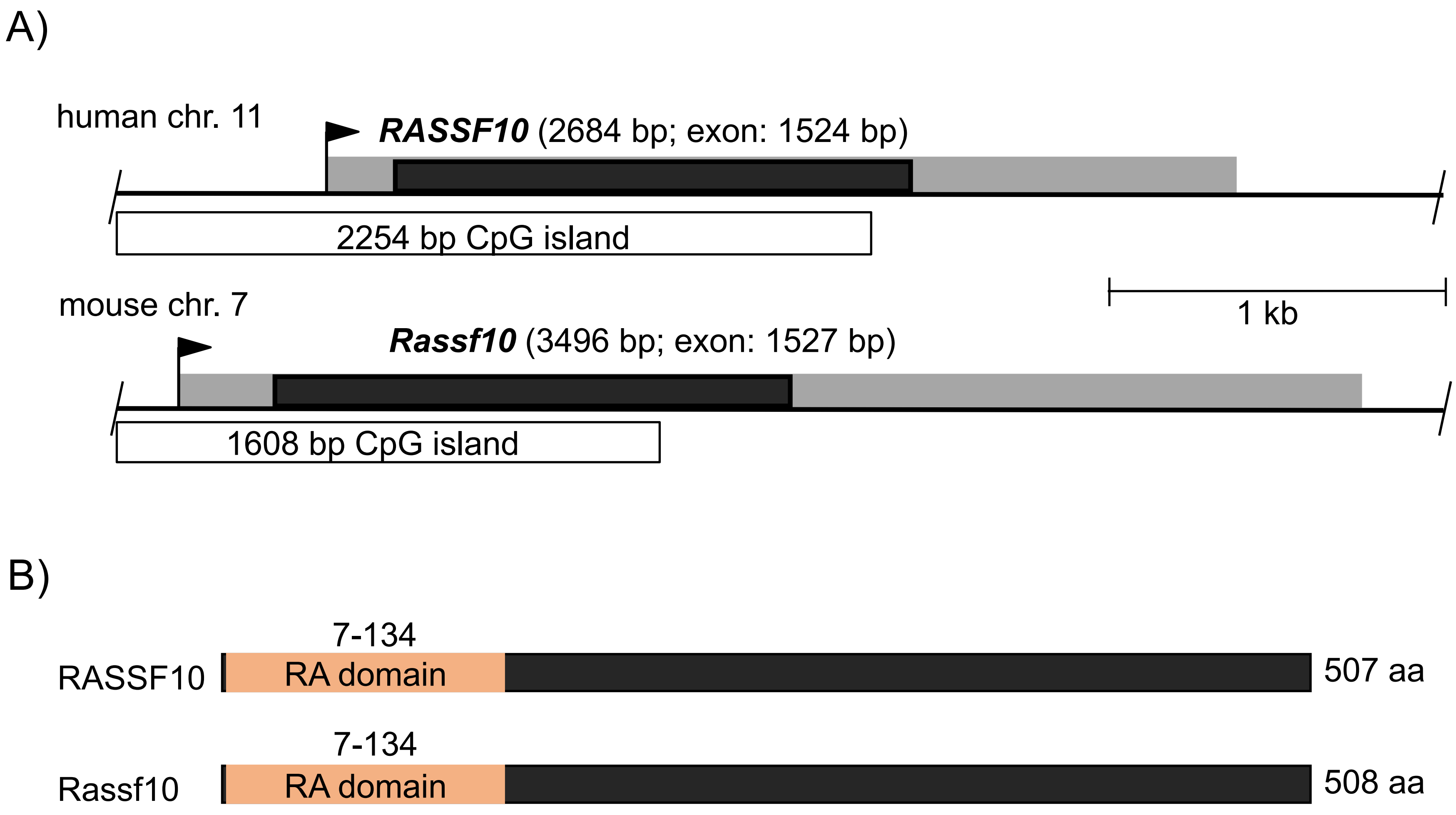

Supplementary Fig. S1: Comparison of human RASSF10 and mouse Rassf10. **A)** Genomic structure with CpG island, coding sequence and exon (black). **B)** The expressed protein contains an N-terminal RAS-association domain (RA domain).

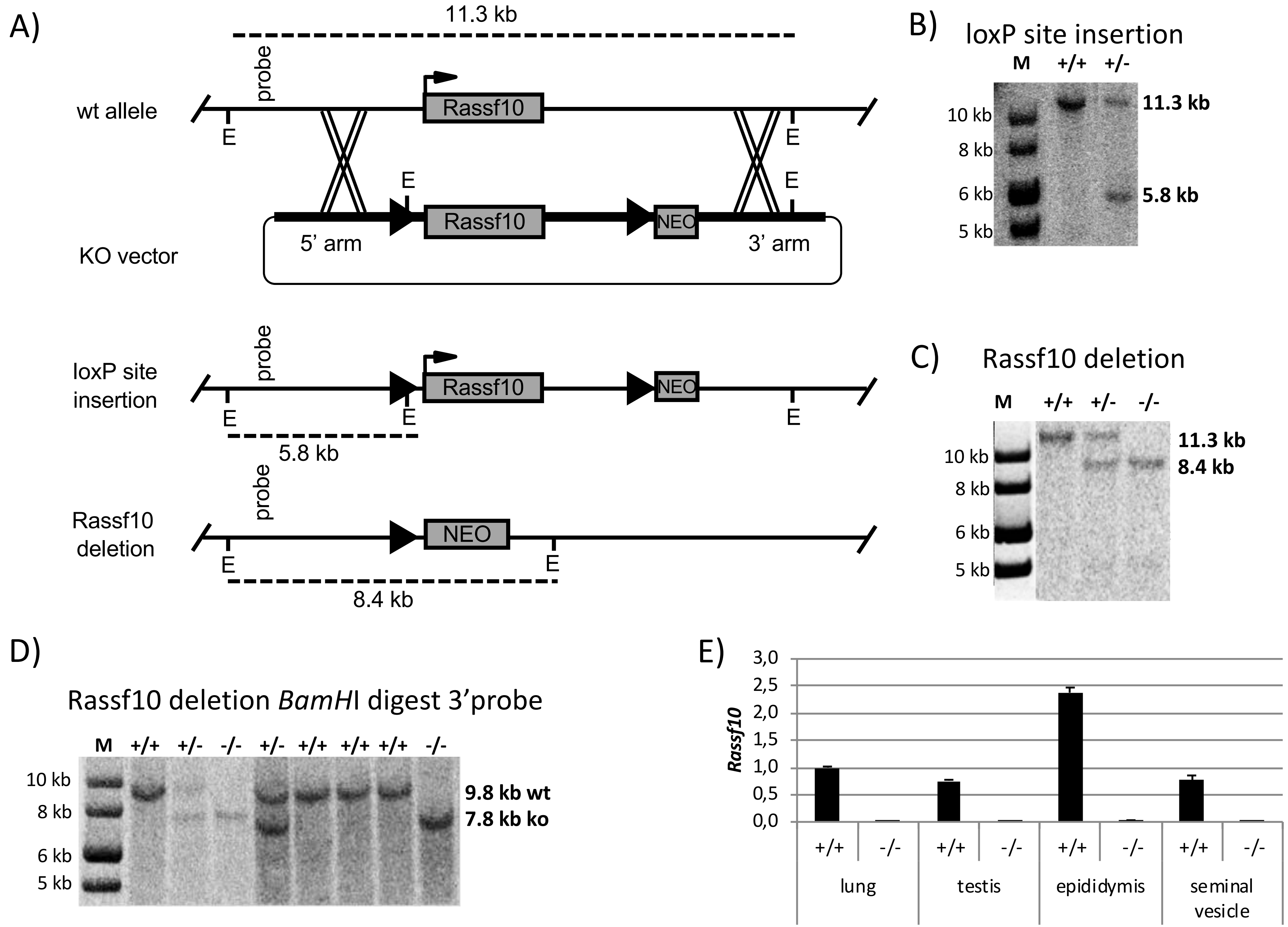

Supplementary Fig. S2: Verification of genomic knockout of Rassf10 by Southern Blotting. A) Schematic procedure of Rassf10 deletion from generation of KO vector, to homozygous recombination of modified Rassf10 construct in embryonic stem cells of 129/C57, recombination and deletion of Rassf10 by Cre Recombinase (loxP based) to final deletion of genomic Rassf10 site. 5'arm and 3'arm are regions for site directed homologous recombination of cloned Rassf10 fragment (carrying loxP sites and Neo cassette) and was performed in embryonic stem cells. Neo (neomycin resistance) allowed positive selection of successful homologous recombination. LoxP represents floxed sequences directing CRE recombinase to site action for deletion of Rassf10. **B)+C)** Southern blotting of Rassf10-knockout animals for verifying successful Rassf10 knockout. Genomic DNA for genotyping was isolated from mice tails and subsequently *EcoR*I digested. Digestion products were separated on 0.8% TAE gel and blotted onto nylon membrane. A probe radioactively marked by P^{32} and directed against 5'region of Rassf10 detected wt-Rassf10, floxed Rassf10 and heterozygous/homozygous knockout of Rassf10 by phosphor imager. The wt-Rassf10 fragment (+/+) is 11.325 bp and the floxed Rassf10 allele is 5.812 bp, due to an additional *EcoR*I site. Interbreeding floxed Rassf10 mice with CRE recombinase expressing mice led to deletion of Rassf10 and loss of additional *EcoR*I site. The Rassf10 knockout DNA fragment is a size

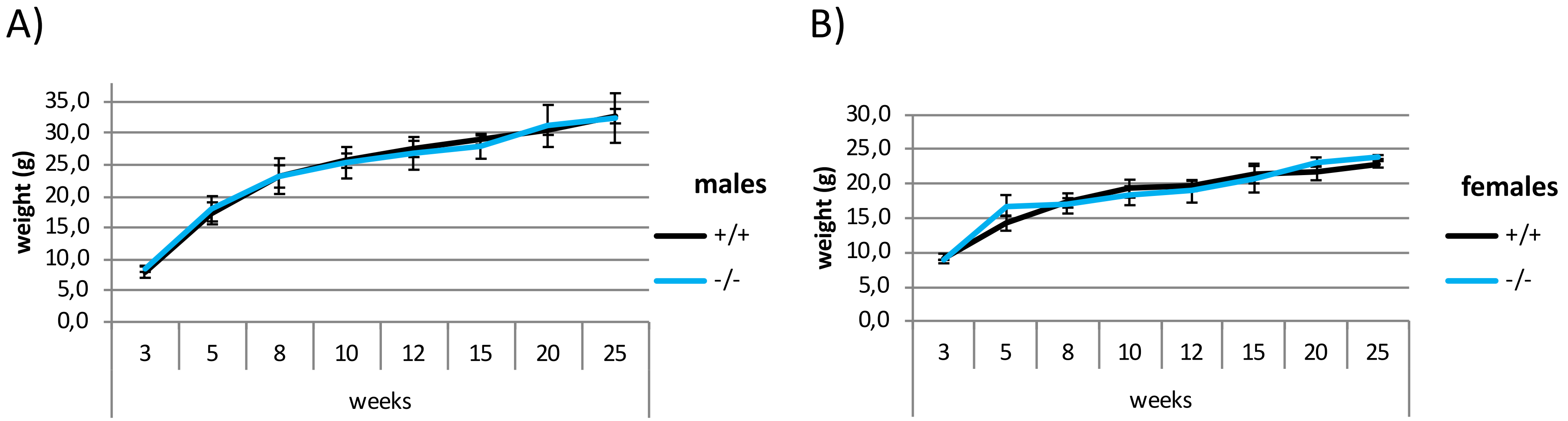

Supplementary Fig. S3: Weight curve of developing Rassf10 knockout mice. Male **A)** and female **B)** Rassf10 knockout mice and according wt littermates were monitored for 25 weeks from 3 weeks onward. Mean weight and SD are shown. Animal numbers are Rassf10$^{+/+}$ males n=11, Rassf10$^{-/-}$ males n=8, Rassf10$^{+/+}$ females n=11 and Rassf10$^{-/-}$ females n=7.

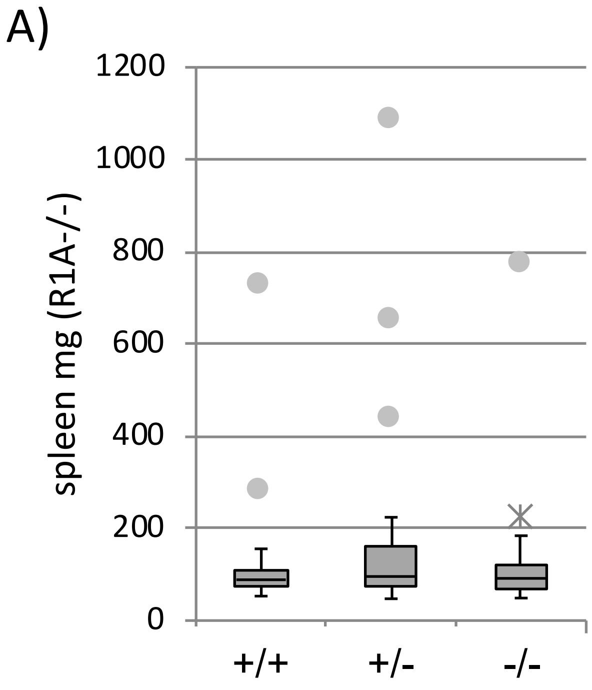

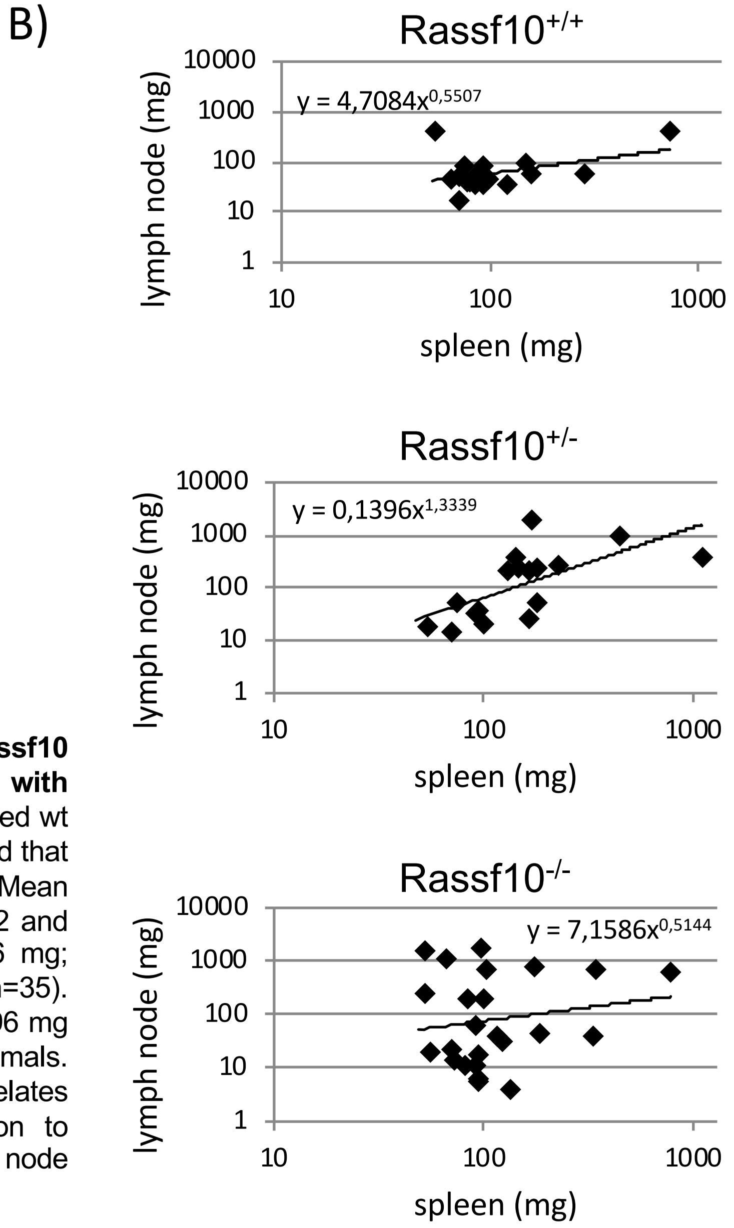

Supplementary Fig. S4: Spleen enlargement in Rassf10 knockout mice (RASSF1A/R1A$^{-/-}$) correlated with increased lymph nodes. A) Spleen weight of sacrificed wt or Rassf10 knockout mice was determined and showed that loss of Rassf10 led to an increase in spleen weight. Mean spleen weight of wt-Rassf10 mice is 91±30 mg; n=12 and mean spleen weight of Rassf10$^{+/-}$R1A$^{-/-}$ is 163±176 mg; n=34) and for Rassf10$^{-/-}$R1A$^{-/-}$ is 129±202 mg; n=35). Median spleen weight is 86 mg for Rassf10$^{+/+}$R1A$^{-/-}$, 96 mg for Rassf10$^{+/-}$R1A$^{-/-}$ and 92 mg for Rassf10$^{-/-}$R1A$^{-/-}$ animals. **B)** Spleen weight increase after Rassf10 loss correlates with presence of enlarged lymph nodes in relation to wildtype animals. Results are depicted as log10 lymph node weight (mg) vs. log10 spleen weight (mg).

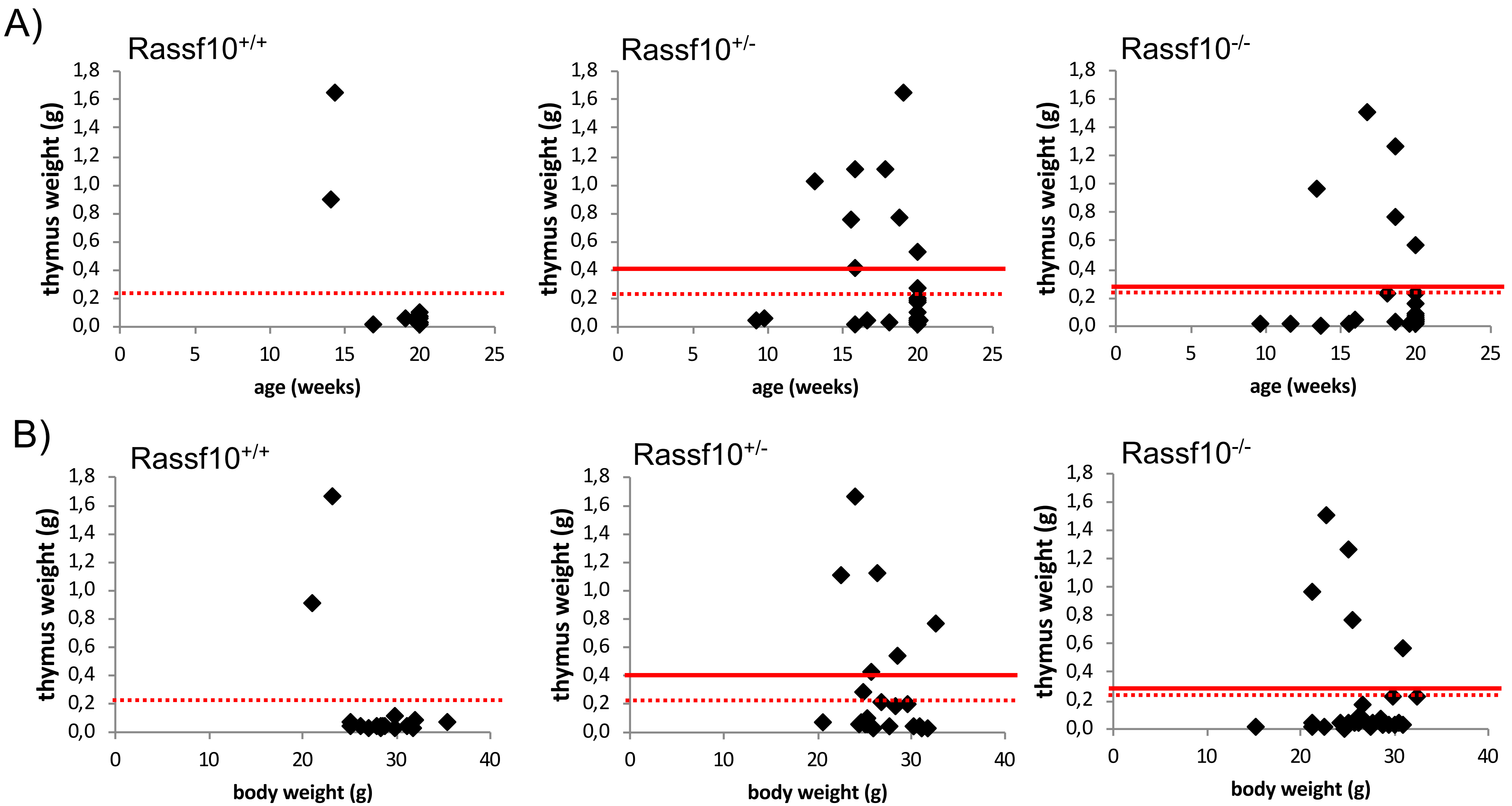

Supplementary Fig. S5: Thymus enlargement in Rassf10 knockout mice (p53$^{-/-}$). Thymus weight of sacrificed wt or Rassf10 knockout mice was determined and plotted relative to age **A)** and body weight **B)**. Mean thymus weight of wt-Rassf10 mice (0.185 g; n=18) is shown as dotted red line and mean thymus weight of either Rassf10$^{+/-}$p53$^{-/-}$ (0.345 g; n=26) or Rassf10$^{-/-}$p53$^{-/-}$ (0.201 g; n=33) is depicted as red line. Thymus weight is increased after Rassf10 loss and irrespective of age and body weight.

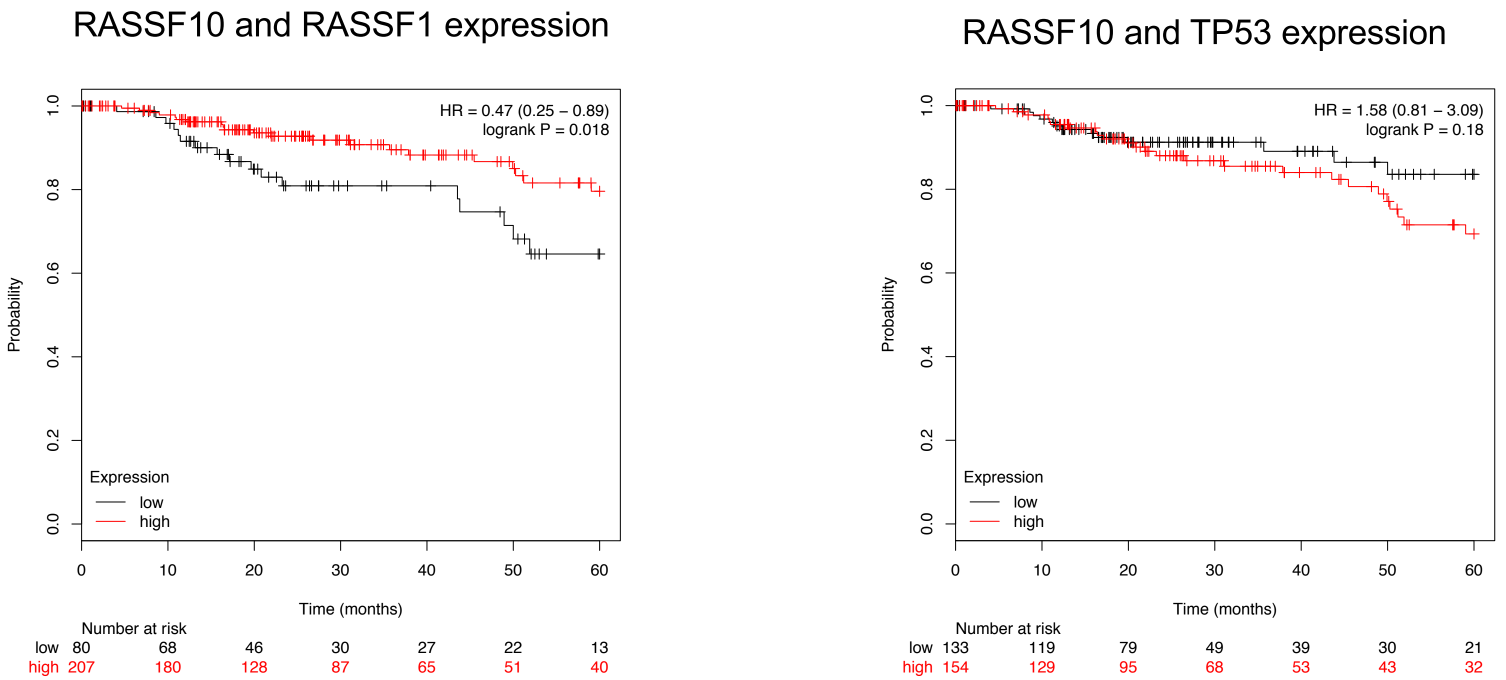

Supplementary Fig. S6: Overall survival rate by combined expressional analysis of RASSF10 and RASSF1 or TP53 in kidney renal cell papillary carcinoma. Mean expression of RASSF10 and RASSF1 or TP53 in renal papillary cell carcinoma (n=288) was analyzed in the pan-cancer RNA seq panel with Kaplan-Meier Plotter[42] and survival probability was plotted for low (black) expressing and high (red) expressing probes.

Supplementary Table S1. Mutation frequency for *RASSF10*, *RASSF1* and *TP53*

cancer	gene	genomic alterations
primary (TCGA)	*RASSF10*	none
	RASSF1	< 2% (across cancer types)
	TP53	80 % lung, 70 % head-neck, 50% colorectal, 30% breast, 30% kidney chromo., 5 % kidney clear cell

Supplementary Table S2. Primer list

	gene		Sequence (upper and lower primer)
RT-primers	*ß-ACTIN*		CCTTCCTTCCTGGGCATGGAGTC
			CGGAGTACTTGCGCTCAGGAGGA
	GAPDH		TGGAGAAGGCTGGGGCTCAT
			GACCTTGGCCAGGGGTGCTA
	RASSF10		GCGCCATGGATCCTTCGGAAAA
			GGCAGCGCCTCGTCGTCGTCCT
	ß-Actin		CCACCATGTACCCAGGCATT
			AGGGTGTAAAACGCAGCTCA
	Gapdh		GCCGCCTGGAGAAACCTGCC
			CCCCGGCATCGAAGGTGGAA
	Rassf10		CTCGCGAACCTCAGCCTCAGACC
			GGGATTCACACACAGGGGGTACCG
CoBRA primers (nested)	*RASSF10*		ATAAGTAGAGGAGTTAGTAGGTTAAAGGAGA
			AAATACAAAAAACTCAAAACCCAAACCC
			GTGGAGGGATTTTTGAATTTTTTTT
			AAATACAAAAAACTCAAAACCCAAACCC

Supplementary Table S3. RASSF10 guides

Guides in px549	Upper oligo (5‘-3‘)	Lower oligo (5‘-3‘)
Guide 1	CACCGCGGAAAAGAAGATATCGGTG	AAACCACCGATATCTTCTTTTCCGC
Guide 2	CACCGTCCTCGTCGTCCTCGTTCGG	AAACCCGAACGAGGACGACGAGGAC
Guide 3	CACCGCAGGAAGAGAAGCTGGTGTC	AAACGACACCAGCTTCTCTTCCTGC
Guide 4	CACCGCGGAGCCGGCGGCTGGGGT	AACACCCCAGCCGCCGGCTCCGC
