# Supplementary material for: RASSF10 is frequently epigenetically inactivated in kidney cancer and its knockout promotes neoplasia in cancer prone mice
Source: Oncogene. 2020 Feb 11;39(15):3114–27. doi: 10.1038/s41388-020-1195-6 (PMC7142015; doi:10.1038/s41388-020-1195-6)
Supplement: Supplementary file 2 — Supplementary datasets [file 41388_2020_1195_MOESM2_ESM.pdf]

Supplementary information of datasets and software used in this study.

Analysis performed using the R2[1] *Genomics Analysis and Visualization Platform*:

Figure 4: RASSF10 expression across human tissues, Normal Tissues GTeX v4 - GTeX - 2921 - RPKM - ensgtexv4, Available on R2 since: 2015-09-17, RASSF10 (ENSG00000189431.5) APS=1.8101(2032) Avg=1.6, Transform: 2log, Sample Filter Select track: tissue; Methylation Expression Correlation: RASSF10, Dataset Project: TCGA, Data Type: 450k Methylation Array 450k Infinium chip, Select: cg05817758, beta value and log2 (normalized rsem+1) of RASSF10, Correlation method: Spearman, Fit linear regression, for KIRC Kidney renal clear cell carcinoma, KIRP Kidney renal papillary cell carcinoma and Breast invasive carcinoma. Analysis performed using the KM Plotter[2-5].

Figure 5: Mixed Renal - Kort - 79 - MAS5.0 - u133p2, Source: GEO ID: gse11024 Dataset Date: 2008-06-05, RASSF10 (238755_at) APS=101(71) Avg=92.6, Clinisnitch: subgroup significant association, Sample Filter Select track: subgroup; Tumor Kidney Chromophobe - TCGA - 66 - rsem - tcgars, Source: TCGA ID: KICH, Available on R2 since: 2015-12-11, RASSF10 (RASSF10_644943) APS=52.9837(63) Avg=50.6, and Tumor Kidney Renal Clear Cell Carcinoma - TCGA - 533 - rsem - tcgars, Source: TCGA ID: KIRC, Available on R2 since: 2015-12-11, RASSF10 (RASSF10_644943) APS=12.213(479) Avg=11.0, Clinisnitch: ajcc_pathologic_tumor_stage, Sample Filter Select track: ajcc_pathologic_tumor_stage; Disease Kidney (ADPKD) - Pei - 21 - MAS5.0 - u133p2, Source: GEO ID: gse7869 Dataset Date: 2009-08-14, RASSF10 (238755_at) APS=173.27(21) Avg=173.3, Clinisnitch: type, Sample Filter Select track: type. Gene: RASSF10, Dataset Project: TCGA, Data Type: Illumina HiSeq RNAseq, Normal/Tumor and log2(normalized rsem+1), for KICH Kidney Chromophobe, KIRP Kidney renal papillary cell carcinoma and KIRC Kidney renal clear cell carcinoma. Analysis performed using MethSurv[6]. TCGA cancer datasets: KIRP Kidney renal papillary carcinoma and KIRC Kidney renal clear cell carcinoma, Relation to island: Island, Genomic Region: TSS200: CpG site: cg05817758, Split by: best maxstat.

References

1. Molenaar JJ, Koster J, Zwijnenburg DA*, et al.* Sequencing of neuroblastoma identifies chromothripsis and defects in neuritogenesis genes. *Nature* 2012; **483:** 589-593.

2. Nagy A, Lanczky A, Menyhart O*, et al.* Validation of miRNA prognostic power in hepatocellular carcinoma using expression data of independent datasets. *Sci Rep* 2018; **8:** 9227.

3. Szasz AM, Lanczky A, Nagy A*, et al.* Cross-validation of survival associated biomarkers in gastric cancer using transcriptomic data of 1,065 patients. *Oncotarget* 2016; **7:** 49322-49333.

4. Gyorffy B, Surowiak P, Budczies J*, et al.* Online survival analysis software to assess the prognostic value of biomarkers using transcriptomic data in non-small-cell lung cancer. *PLoS One* 2013; **8:** e82241.

5. Gyorffy B, Lanczky A, Eklund AC*, et al.* An online survival analysis tool to rapidly assess the effect of 22,277 genes on breast cancer prognosis using microarray data of 1,809 patients. *Breast Cancer Res Treat* 2010; **123:** 725-731.

6. Modhukur V, Iljasenko T, Metsalu T*, et al.* MethSurv: a web tool to perform multivariable survival analysis using DNA methylation data. *Epigenomics* 2018; **10:** 277-288.
